# Sorting living mesenchymal stem cells using a TWIST1 RNA-based probe depends on incubation time and uptake capacity

**DOI:** 10.1007/s10616-019-00355-w

**Published:** 2019-11-14

**Authors:** Chantal Voskamp, Jeroen van de Peppel, Simona Gasparini, Paolo Giannoni, Johannes P. T. M. van Leeuwen, Gerjo J. V. M. van Osch, Roberto Narcisi

**Affiliations:** 1 Department of Orthopaedics, Erasmus MC, 3015 CN Rotterdam, The Netherlands; 2 Department of Internal Medicine, Erasmus MC, Rotterdam, The Netherlands; 3 Department of Experimental Medicine, University of Genova, Genoa, Italy; 4 Department of Otorhinolaryngology, Erasmus MC, 3015 CN Rotterdam, The Netherlands

**Keywords:** Mesenchymal stem cells (MSCs), TWIST1, RNA probes, Tissue engineering, Cell sorting, Expansion

## Abstract

Bone marrow derived mesenchymal stromal cells (BMSCs) are multipotent progenitors of particular interest for cell-based tissue engineering therapies. However, one disadvantage that limit their clinical use is their heterogeneity. In the last decades a great effort was made to select BMSC subpopulations based on cell surface markers, however there is still no general consensus on which markers to use to obtain the best BMSCs for tissue regeneration. Looking for alternatives we decided to focus on a probe-based method to detect intracellular mRNA in living cells, the SmartFlare technology. This technology does not require fixation of the cells and allows us to sort living cells based on gene expression into functionally different populations. However, since the technology is available it is debated whether the probes specifically recognize their target mRNAs. We validated the TWIST1 probe and demonstrated that it specifically recognizes TWIST1 in BMSCs. However, differences in probe concentration, incubation time and cellular uptake can strongly influence signal specificity. In addition we found that *TWIST1*^*high*^ expressing cells have an increased expansion rate compared to *TWIST1*^*low*^ expressing cells derived from the same initial population of BMSCs. The SmartFlare probes recognize their target gene, however for each probe and cell type validation of the protocol is necessary.

**Electronic supplementary material:**

The online version of this article (10.1007/s10616-019-00355-w) contains supplementary material, which is available to authorized users.

## Introduction

Multipotent progenitor cells from bone marrow aspirates can differentiate into chondrocytes, osteoblasts and adipocytes (Pittenger et al. 1999). These progenitor cells, often referred to as bone marrow-derived mesenchymal stem or stromal cells (BMSCs), are appealing for cell-based tissue engineering purposes. Unfortunately, their limited expansion capacity and their heterogeneity, hinder their clinical use (Banfi et al. 2000; Bonab et al. 2006; Chen et al. 2005; Li et al. 2011). Several studies investigated cell surface molecules to identify specific subpopulations of BMSCs (Alvarez-Viejo et al. 2015; Buhring et al. 2007; Cleary et al. 2016; Delorme et al. 2008; Sacchetti et al. 2007; Sivasubramaniyan et al. 2013). However, despite the great effort, there is still no general consensus on the surface markers that need to be used to define or select the best BMSC subset for tissue engineering. One drawback of surface markers is that their function is often unknown, so alternative markers are necessary to select cells according to their function (Clevers and Watt 2018).

Recently, a probe-based method to detect intracellular mRNA in living single cells has been developed, the SmartFlare technology (Seferos et al. 2007; Prigodich et al. 2009). The SmartFlare technique is a promising tool to sort BMSCs into functionally different populations. The SmartFlare probes are taken up by the cells via endocytosis and if the target mRNA is present, the probes bind to the target mRNA and fluorescent reporters are released and detectable (Figure S1A). Since the SmartFlare technology is available, this technique already successfully identified cancer cells (McClellan et al. 2015; Kronig et al. 2015) and pluripotent stem cells (Lahm et al. 2015). Additionally it was applied to investigate a Nodal expressing subpopulation of melanoma cells (Seftor et al. 2014), and to study a subpopulation of human BMSCs with an enhanced osteogenic potential (Li et al. 2016). However, other studies did not find a correlation between the SmartFlare fluorescence intensity and mRNA expression measured by RT-PCR (Czarnek and Bereta 2017; Yang et al. 2018). In addition, Czarnek et al. (2017) found that the SmartFlare signal intensity correlates with the probe uptake ability of the cells.

To assess if the SmartFlare technique can be used to sort different populations of BMSCs based on gene expression, we focused on the validation of a probe for *TWIST1*. TWIST1 is a transcription factor that is involved in the regulation of BMSC proliferation (Goodnough et al. 2012; Isenmann et al. 2009; Tian et al. 2015) and differentiation (Isenmann et al. 2009; Boregowda et al. 2016; Cleary et al. 2017; Narcisi et al. 2015, 2016). In the present study, we evaluated the SmartFlare protocol in order to detect a specific probe signal in our culture conditions and illustrated that the SmartFlare fluorescence intensity is associated with probe concentration, incubation time and cellular uptake capacity.

## Materials and methods

### Isolation and culture of human adult bone marrow mesenchymal stem cells

Human adult bone marrow aspirates were obtained from femoral biopsies of 8 patients (22–79 years) undergoing total hip replacement (MEC 2015-644, MEC 2004-142: Erasmus Medical Center, Rotterdam; MEC 2011.07 Albert Schweitzer Hospital, Dordrecht), after obtaining informed consent and full ethical approval by the Erasmus MC and Albert Schweitzer ethics committee.

Human BMSCs were isolated, seeded at the density of 2300 cells/cm^2^ and cultured as previously described in standard expansion media, containing 10% FCS (Lonza, Verviers, Belgium; selected batch:1S016) and 1 ng/ml FGF2 (AbD Serotech, Kidlington, United Kingdom) (Narcisi et al. 2016). The medium was refreshed twice a week. BMSCs expanded for 3 to 6 passages were used for experiments.

### SmartFlare probes

Cells were treated with the SmartFlare probe when they were sub-confluent. SmartFlare probes TWIST1-Cy3 (the only label available for TWIST1), Uptake-Cy5, and GAPDH-Cy5 were purchased from Merck. The probes were resuspended in 50 µl sterile nuclease free water, 1:20 prediluted in PBS (Lonza) and added to the cells with a final concentration of 50 pM or 100 pM. The cells were incubated for 6 or 16 h at 37 °C and 5% CO_2_ and analyzed using flow cytometry. To assure a broad range of *TWIST1* gene expression during the validation of the TWIST1-Cy3 probe, BMSCs from two different donors were mixed and treated with the TWIST1-Cy3 probe.

### Flow cytometry and FACS

Flow cytometry analysis was performed using a BD Fortessa and the data was analyzed using FlowJo V10 software. The cells were sorted using a BD Biosciences FACS Aria and the data was analyzed using BD FACS Diva 8.0.1 software. Cell debris were excluded from the population through forward scatter (FSC)/side scatter (SSC) gate and doublets were excluded using FSC-A/FSC-H gate (Figure S2A). To confirm effective sorting, the sorted populations were reanalyzed (Figure S2B). Mean fluorescent intensity (MFI) was measured using FlowJo V10 software. The two different gates *TWIST1*^*high*^ and *TWIST*^*low*^ were established based on the TWIST1-Cy3 fluorescence intensity, 15–25% of the extremes or two different gates *TWIST1/Uptake*^*high*^ and *TWIST1/Uptake*^*low*^ were established based on the TWIST1-Cy3 fluorescence intensity, 15% of the extremes with a comparable Uptake-Cy5 fluorescence intensity. The sorted cells were collected in PBS with 1% FCS and reseeded with a density of 2300 cells per cm^2^ or used for RNA isolation.

### Real time PCR analysis

Post-sorting, 200,000 BMSCs per sample were spun down and treated on ice with RLT lysis buffer (Qiagen, Hilden, Germany) with 1% β-mercaptoethanol. BMSCs in monolayer were washed with PBS and treated on ice with RLT lysis buffer (Qiagen) with 1% β-mercaptoethanol. A range of 0.25–1.00 µg of purified RNA (RNeasy Micro Kit; Qiagen) was reverse transcribed into cDNA (RevertAid First Strand cDNA Synthesis Kit; MBI Fermentas, St. Leon-Rot, Germany). RT-PCR was performed using an annealing temperature of 60 °C on a C1000 Touch™ Thermal Cycler using SybrGreen (Eurogentec, Seraing, Belgium). The data were normalized to the housekeeper gene *RSP27a*. The relative expression was calculated according to the 2^−ΔΔCt^ formula. The primers used for RT-PCR are listed in (Table S1).

### Data analysis

Linear correlation (Fig. 2c) was analyzed with GraphPad Prism Software 5.00 assuming normal distribution of the data.

## Results

### TWIST1 SmartFlare detects *TWIST1* mRNA after 6 h using a concentration of 50 pM in human BMSCs

**Fig. 1:**
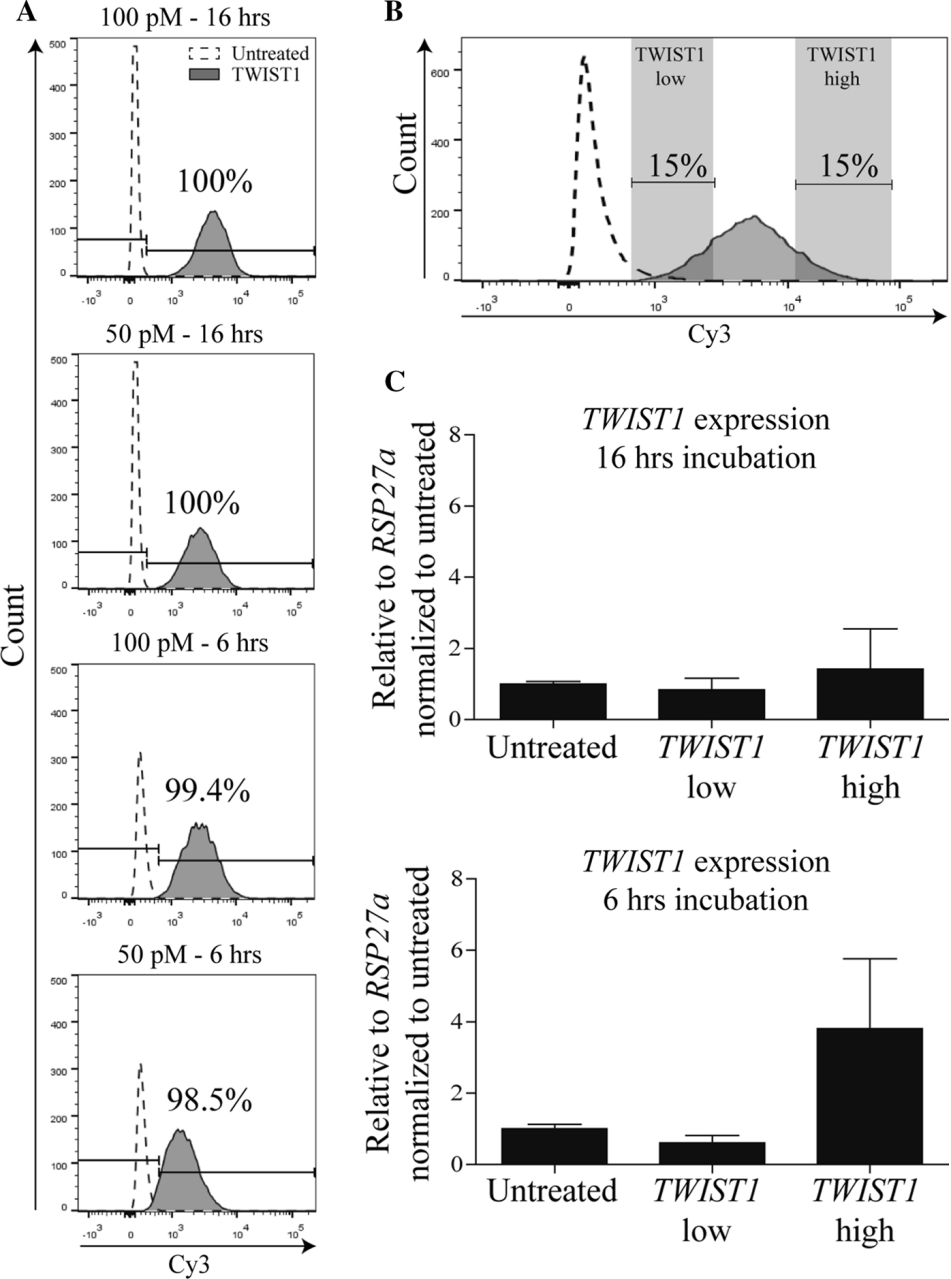
TWIST1 SmartFlare probes are efficiently taken up by BMSCs after 6 h. **a** Flow cytometry histogram of untreated BMSCs and BMSCs with 100 pM or 50 pM TWIST1-Cy3 probe incubated for 16 or 6 h,  % shows percentage Cy5 positive cells. **b** Gating strategy based on TWIST1-Cy3 intensity. The doted graph represents unstained BMSCs and the gray graph represents BMSCs with TWIST1-Cy3 probes. **c** BMSCs were sorted based on TWIST1-Cy3 intensity after 16 and 6 h of probe incubation. TWIST1 transcripts were analysis by RT-PCR. Values represent the mean ± SD from duplicates or quadruplicate

SmartFlare probes enter the cell via endocytosis and this process can vary between different cell types (Choi et al. 2013). The probe incubation time and concentration which is suggested by the manufacturer is 16 h and 100 pM. However we also included a 6 h timepoint and a concentration of 50 pM in order to verify whether or not it was possible to further optimize the SmartFlare protocol for TWIST1 in BMSCs. Interestingly, already after 6 h with a probe concentration of 50 pM, 98.5% of the cells were positive for TWIST1 SmartFlare signal (Fig. 1a; lowest panel). No major differences in SmartFlare signal intensity were observed between the different probe concentrations and incubation times (Fig. 1a).

To study TWIST1-Cy3 signal specificity, BMSCs were treated with TWIST1-Cy3 probe for 16 h or 6 h, sorted based on the TWIST1-Cy3 signal by FACS and subsequently tested by RT-PCR. Our FACS gating strategy consisted of sorting 15% of the BMSCs with the lowest TWIST1-Cy3 signal and 15% of the BMSCs with the highest TWIST1-Cy3 signal (*TWIST1*^*low*^ vs *TWIST1*^*high*^; Fig. 1b). To our surprise no difference in relative *TWIST1* gene expression was detected between *TWIST1*^*low*^ and *TWIST1*^*high*^ cells after 16 h of probe incubation (Fig. 1c). This indicates that although we observe a TWIST1 SmartFlare signal after 16 h, this signal is probably not specific for *TWIST1* gene expression. However after 6 h incubation we confirmed that *TWIST1*^*high*^ BMSCs have a higher *TWIST1* gene expression than the *TWIST1*^*low*^ population (6.25-fold difference; Fig. 1c). These data shows that the TWIST1 probe specifically detects *TWIST1* gene expression in this population of BMSCs already after 6 h incubation with a concentration of 50 pM probe. In addition we observed that more than 97.3% of cells were positive for the Uptake control probe, a probe which is always fluorescent without binding to a target (Supplementary Figure 1B), with 50 pM after 6 h of incubation.

**Fig. 2:**
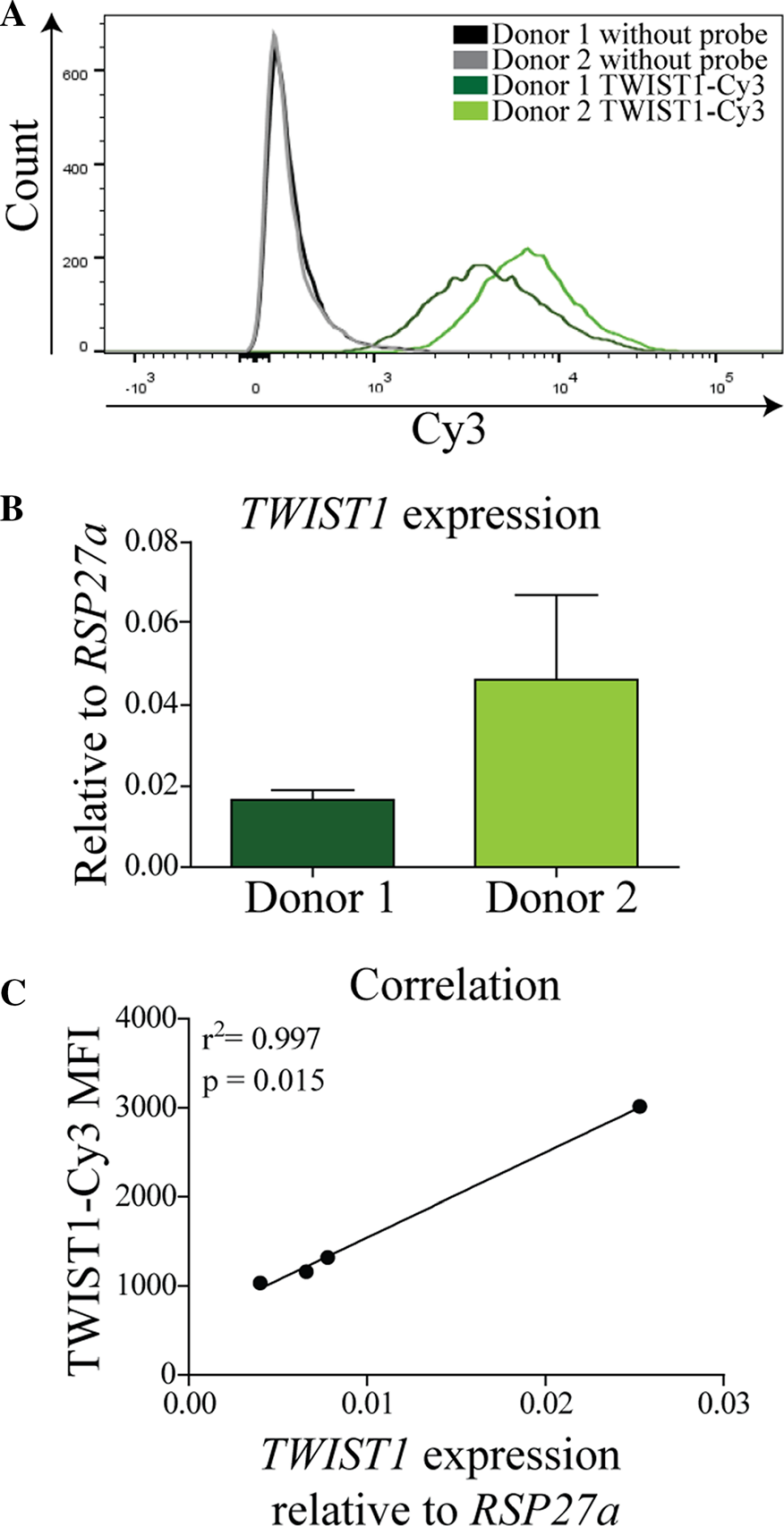
TWIST1 SmartFlare detects TWIST1 mRNA expression. **a** Flow cytometry histogram of BMSCs from two donors untreated or treated with the TWIST1-Cy3 probe for 6 h. **b** TWIST1 RT-PCR results, values represent the mean ± SD from triplicates. **c** Correlation between *TWIST1* expression measured by RT-PCR and TWIST1-Cy3 MFI. Dots represent different donors (N = 4)

To further determine the signal specificity of the TWIST1 probe after 6 h, we analyzed the correlation between the TWIST1-Cy3 signal intensity and *TWIST1* expression by RT-PCR. TWIST1 probe signal intensity from two BMSC populations (referred to as donor 1 and donor 2) was measured using flow cytometry, showing a higher intensity in donor 2 (8775 vs 5645 MFI; Fig. 2a). Transcript analysis confirmed the difference in *TWIST1* expression between the two donors, showing a higher expression in donor 2 (Fig. 2b). We therefore repeated the analysis in four other donors showing a positive and consistent correlation between TWIST1-Cy3 probe intensity and *TWIST1* gene expression (r^2^ = 0.997; Fig. 2c). These data again confirms that the TWIST1 probe specifically targets the *TWIST1* mRNA after 6 h of incubation.

### Correction for cellular probe uptake improves *TWIST1* gene detection

When we repeated the sorting experiment with other donors not always differences in *TWIST1* expression by RT-PCR were observed between *TWIST1*^*low*^ and *TWIST1*^*high*^ sorted cells (Figure S4). Given that, and considering that Czarnek et al. recently showed that uptake capacity can influence the SmartFlare signal specificity (Czarnek and Bereta 2017), we decided to carefully monitor uptake in our BMSC populations.

**Fig. 3:**
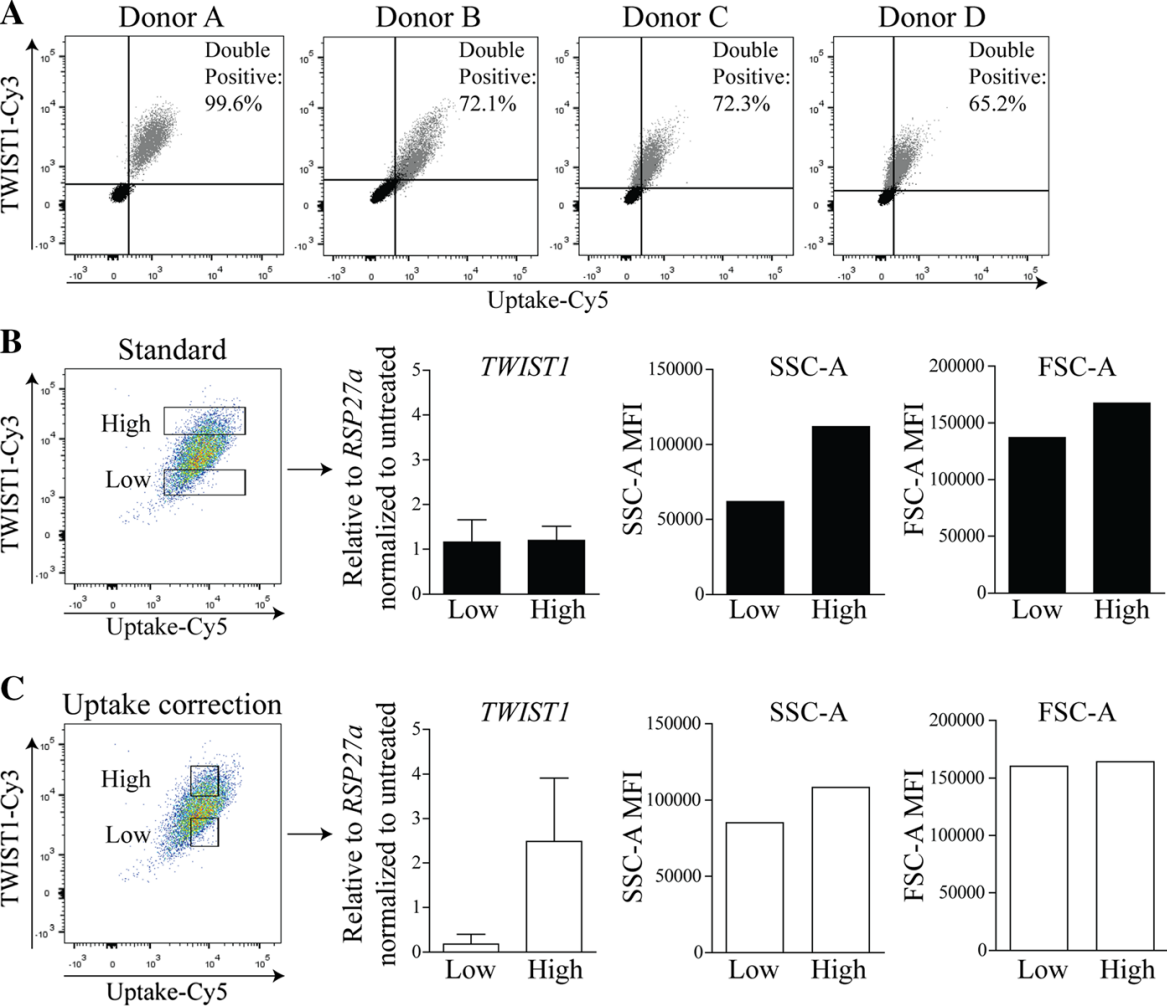
Correction for cellular probe uptake improves TWIST1 gene detection. **a** Flow cytometry plots of BMSCs of four donors treated with both TWIST1-Cy3 and Uptake-Cy5 probe for 6 h (grey). The perpendicular lines represent the unstained control (black) for each donor.  % shows percentage Cy3 and Cy5 double positive cells. **b**, **c** FACS gating strategies using TWIST1-Cy3 and Uptake-Cy5 probes for 6 h and TWIST1 RT-PCR results, values represent the mean ± SD from duplicates. SSC-A MFI and FSC MFI of Standard and Uptake correction low vs high

To evaluate the effect of cellular uptake on the TWIST1 signal, BMSCs from 4 different donors were double labeled with TWIST1-Cy3 and Uptake-Cy5 probes (Figure S1B). At least 65% of the BMSCs were able to take up both the TWIST1-Cy3 and Uptake-Cy5 probe (Fig. 3a) and we demonstrated that BMSCs from different donors have a different uptake capacity (Figure S5). Moreover, it is clear from the FACS analysis that there is a general positive correlation between Uptake-Cy5 signal and TWIST-Cy3 signal (the higher the TWIST1 signal, the higher the Uptake signal), although with variation between donors (Fig. 3b and Figure S5). This indicates that in BMSCs from different donors the TWIST1-Cy3 signal can be affected by the cellular uptake capacity, with a degree that depends on the individual uptake capacity of the cells in the BMSC population. To determine whether or not the detected differences in cellular uptake have an effect on *TWIST1* gene detection, BMSCs with a high variation in Uptake-Cy5 fluorescence intensity were treated with both TWIST1-Cy3 and Uptake-Cy5 probes and were sorted by FACS using two different sorting strategies or left unsorted. In the first gating strategy, similar to that previously used, 15% of the BMSCs with the lowest TWIST1-Cy3 signal and 15% of the BMSCs with the highest TWIST1-Cy3 signal (*TWIST1*^*high*^) were sorted (Fig. 3c; left panel). In the second gating strategy we corrected for the uptake signal (Fig. 3d; left panel) by sorting *TWIST1*^*high*^ and *TWIST1*^*low*^ cells with a minimal uptake variation. Gene expression analysis showed no differences between *TWIST1*^*low*^ and *TWIST1*^*high*^ populations in the absence of uptake correction (Fig. 3c; left middle panel), while a strong difference (13.3-fold) was detected between the subpopulations where the TWIST1 signal was corrected for the uptake (Fig. 3d; left middle panel). These data indicate that differences in cellular uptake can strongly influence *TWIST1* detection using SmartFlare. In addition, we observed that the sorted populations of BMSCs corrected for cellular uptake had a similar cellular granularity (Fig. 3c, d; right middle panel) and cell size (Fig. 3c, d; right panel) compared to the populations sorted without uptake correction.

### *TWIST1*^*high*^ BMSCs have a high expansion capacity

**Fig. 4:**
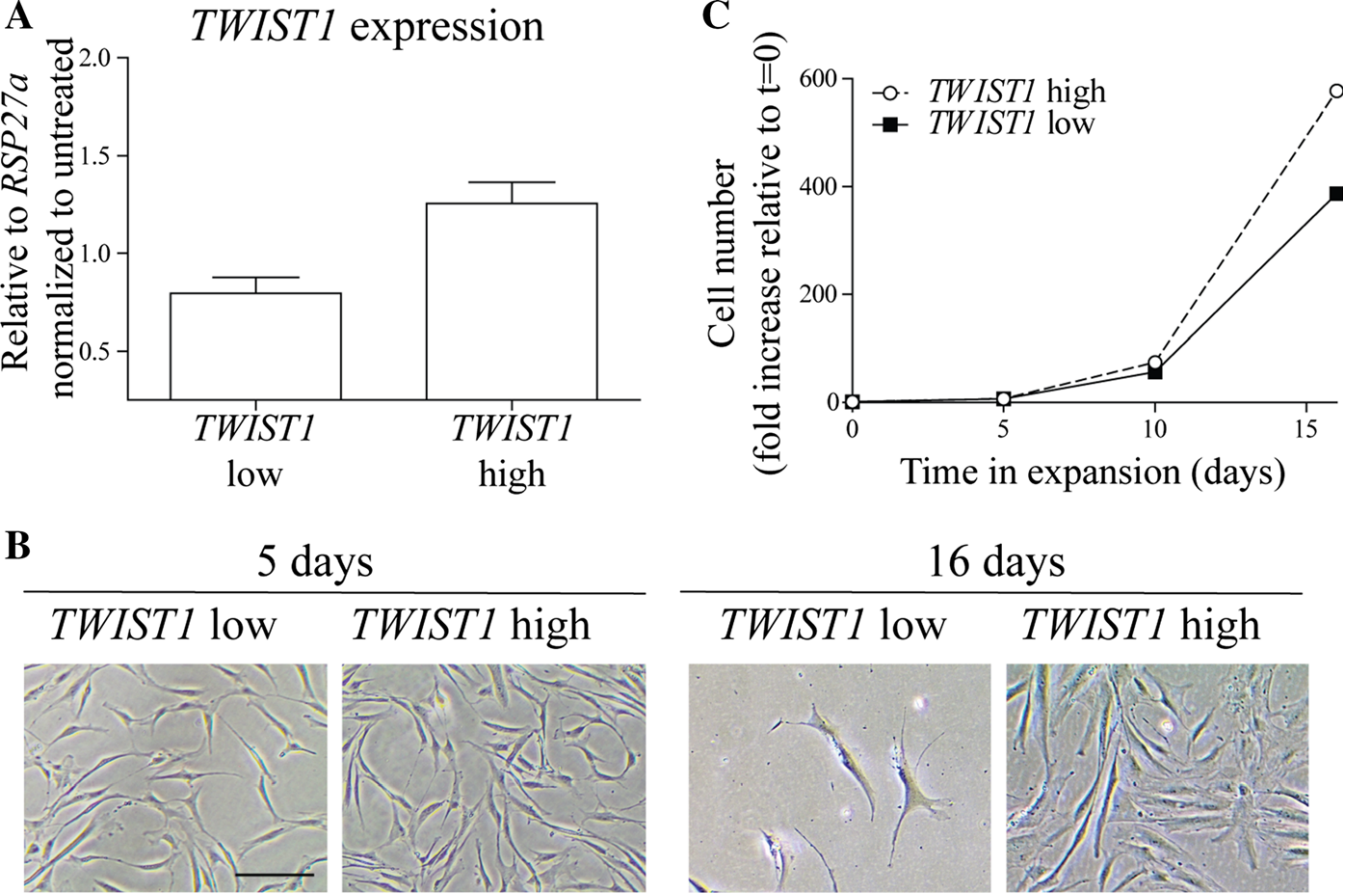
*TWIST1*^*high*^ BMSCs have a high proliferation capacity. **a***TWIST1* RT-PCR results of Untreated, *TWIST1*^*low*^ and *TWIST1*^*high*^ populations, values represent the mean ± SD from duplicates. **b** Morphology of BMSCs 5 days and 16 days after being sorted. Scale bar represents 100 µm. **c** Cell numbers relative to t = 0 of Untreated, *TWIST1*^*low*^ and *TWIST1*^*high*^ were passaged and counted on day 0, day 5, day 10 and day 16

In order to further validate our sorting strategy and prove for the first time the pro-proliferative role of *TWIST1* in a subpopulation of BMSCs, we sorted *TWIST1*^*high*^ and *TWIST1*^*low*^ cells and we compared their expansion capacity post-sorting. RT-PCR confirmed that *TWIST1*^*high*^ BMSCs had a higher relative *TWIST1* gene expression than *TWIST1*^*low*^ BMSCs (1.6-fold difference; Fig. 4a). No evident differences in morphology between *TWIST1*^*low*^ and *TWIST1*^*high*^ were observed 5 days post sorting, while 16 days post sorting *TWIST1*^*low*^ BMSCs appeared more enlarged compared to the *TWIST1*^*high*^ BMSCs (Fig. 4b). Moreover, *TWIST1*^*high*^ BMSCs showed a higher expansion capacity than the *TWIST1*^*low*^ population (Fig. 4c; 1.5-fold difference after 3 passages) and, 16 days post sorting, the *TWIST1*^*low*^ BMSCs stop growing while the *TWIST1*^*high*^ BMSCs were still expanding (data not shown). This indicates that within a population of BMSCs derived from one donor, the *TWIST1*^*high*^ expressing cells have a higher expansion rate compared to the *TWIST1*^low^ expressing cells.

## Discussion

In this study, we evaluated the use of the SmartFlare technique to detect *TWIST1* expression at a single cell level in living BMSCs. Multiple studies successfully detected mRNA expression with the SmartFlare technique (McClellan et al. 2015; Kronig et al. 2015; Lahm et al. 2015; Seftor et al. 2014; Li et al. 2016). However, two recent studies showed that different SmartFlare probes were not able to specifically detect their target mRNAs in cell lines and monocytes (Czarnek and Bereta 2017; Yang et al. 2018). Here we showed that SmartFlare is an effective tool to detect *TWIST1* gene expression in living BMSCs, but differences in probe concentration, incubation time and cellular uptake can influence the SmartFlare sensitivity and possibly lead to misinterpretation of the results.

We observed that specific detection of *TWIST1* mRNA expression in BMSCs is possible already after 6 h of incubation with a concentration of 50 pM, TWIST1-Cy3 probe. While most of the studies used 16 h (McClellan et al. 2015; Seftor et al. 2014; Li et al. 2016; Czarnek and Bereta 2017) or even a longer incubation time (Kronig et al. 2015; Lahm et al. 2015; Czarnek and Bereta 2017), we were not able to specifically detect *TWIST1* after 16 h incubation (Figure S4). The SmartFlare technology was recently applied in BMSCs (Li et al. 2016), but never for the detection of *TWIST1* expression. In our study a different protocol was needed compared to the RUNX2 and the SOX9 probes used by Li et al. (2016). Possible explanations could be ascribed to differences in culture conditions, origin of BMSCs or binding efficiency of the probe to the target.

In addition, our data indicate that BMSCs can have a high difference in probe uptake. We observed that these differences strongly influence the TWIST1 SmartFlare specificity. This confirms confirms the data previously reported where was shown that SmartFlare intensity was affected by cellular uptake in 293T cells (Czarnek and Bereta 2017). The differences in uptake capacity can be explained by differences in cell cycle stage between the BMSCs, since endocytosis is reduced during mitosis (Fielding et al. 2012). Here, we were able to overcome this problem by correcting *TWIST1* detection for the cellular uptake based on Uptake probe intensity during sorting. Next, we demonstrate that *TWIST1*^*high*^ expressing BMSCs have a higher expansion capacity than *TWIST1*^*low*^ expressing BMSCs derived from the same donor. A population of BMSCs with a high *TWIST1* expression and a high proliferation rate have already been reported by us and others (Isenmann et al. 2009; Cleary et al. 2017; Narcisi et al. 2015). Here, we showed for the first time that within the same population of BMSCs, the subpopulation of *TWIST1*^*high*^ expressing cells have a higher expansion capacity than the *TWIST1*^*low*^ expressing cells. Alternatively to the use of the uptake control, in a previous report the ratio between two functional markers, RUNX2 and SOX9, was applied (Li et al. 2016). This indirect method could also be used, since it would automatically take into account differences in uptake, as these would not change the ratio, but only the intensity of the individual signals.

## Conclusion

In summary, our data indicate that for each probe and cell type, a validation of the SmartFlare protocol is necessary. Giving that, we were able to successfully use the TWIST1 probe to detect *TWIST1* mRNA in living BMSCs and to sort *TWIST1*^*high*^ BMSCs from a heterogeneous population of cells. Overall, we showed that SmartFlare is a promising tool to divide a heterogeneous population of cells based on gene expression in functionally different populations.

## Electronic supplementary material

Below is the link to the electronic supplementary material.

Supplementary material 1 (PDF 458 kb)

## References

[CR1] Alvarez-Viejo M, Menendez-Menendez Y, Otero-Hernandez J (2015). CD271 as a marker to identify mesenchymal stem cells from diverse sources before culture. World J Stem Cells.

[CR2] Banfi A, Muraglia A, Dozin B, Mastrogiacomo M, Cancedda R, Quarto R (2000). Proliferation kinetics and differentiation potential of ex vivo expanded human bone marrow stromal cells: implications for their use in cell therapy. Exp Hematol.

[CR3] Bonab MM, Alimoghaddam K, Talebian F, Ghaffari SH, Ghavamzadeh A, Nikbin B (2006). Aging of mesenchymal stem cell in vitro. BMC Cell Biol.

[CR4] Boregowda SV, Krishnappa V, Haga CL, Ortiz LA, Phinney DG (2016). A clinical indications prediction scale based on TWIST1 for human mesenchymal stem cells. EBioMedicine.

[CR5] Buhring HJ, Battula VL, Treml S, Schewe B, Kanz L, Vogel W (2007). Novel markers for the prospective isolation of human MSC. Ann N Y Acad Sci.

[CR6] Chen J, Sotome S, Wang J, Orii H, Uemura T, Shinomiya K (2005). Correlation of in vivo bone formation capability and in vitro differentiation of human bone marrow stromal cells. J Med Dent Sci.

[CR7] Choi CH, Hao L, Narayan SP, Auyeung E, Mirkin CA (2013). Mechanism for the endocytosis of spherical nucleic acid nanoparticle conjugates. Proc Natl Acad Sci U S A.

[CR8] Cleary MA, Narcisi R, Focke K, van der Linden R, Brama PA, van Osch GJ (2016). Expression of CD105 on expanded mesenchymal stem cells does not predict their chondrogenic potential. Osteoarthr Cartil.

[CR9] Cleary MA, Narcisi R, Albiero A, Jenner F, de Kroon LMG, Koevoet W, Brama PAJ, van Osch G (2017). Dynamic regulation of TWIST1 expression during chondrogenic differentiation of human bone marrow-derived mesenchymal stem cells. Stem Cells Dev.

[CR10] Clevers H, Watt FM (2018). Defining adult stem cells by function, not by phenotype. Annu Rev Biochem.

[CR11] Czarnek M, Bereta J (2017). SmartFlares fail to reflect their target transcripts levels. Sci Rep.

[CR12] Delorme B, Ringe J, Gallay N, Le Vern Y, Kerboeuf D, Jorgensen C, Rosset P, Sensebe L, Layrolle P, Haupl T, Charbord P (2008). Specific plasma membrane protein phenotype of culture-amplified and native human bone marrow mesenchymal stem cells. Blood.

[CR13] Fielding AB, Willox AK, Okeke E, Royle SJ (2012). Clathrin-mediated endocytosis is inhibited during mitosis. Proc Natl Acad Sci U S A.

[CR14] Goodnough LH, Chang AT, Treloar C, Yang J, Scacheri PC, Atit RP (2012). Twist1 mediates repression of chondrogenesis by beta-catenin to promote cranial bone progenitor specification. Development.

[CR15] Isenmann S, Arthur A, Zannettino AC, Turner JL, Shi S, Glackin CA, Gronthos S (2009). TWIST family of basic helix-loop-helix transcription factors mediate human mesenchymal stem cell growth and commitment. Stem Cells.

[CR16] Kronig M, Walter M, Drendel V, Werner M, Jilg CA, Richter AS, Backofen R, McGarry D, Follo M, Schultze-Seemann W, Schule R (2015). Cell type specific gene expression analysis of prostate needle biopsies resolves tumor tissue heterogeneity. Oncotarget.

[CR17] Lahm H, Doppler S, Dressen M, Werner A, Adamczyk K, Schrambke D, Brade T, Laugwitz KL, Deutsch MA, Schiemann M, Lange R, Moretti A, Krane M (2015). Live fluorescent RNA-based detection of pluripotency gene expression in embryonic and induced pluripotent stem cells of different species. Stem Cells.

[CR18] Li Z, Liu C, Xie Z, Song P, Zhao RC, Guo L, Liu Z, Wu Y (2011). Epigenetic dysregulation in mesenchymal stem cell aging and spontaneous differentiation. PLoS ONE.

[CR19] Li B, Menzel U, Loebel C, Schmal H, Alini M, Stoddart MJ (2016). Monitoring live human mesenchymal stromal cell differentiation and subsequent selection using fluorescent RNA-based probes. Sci Rep.

[CR20] McClellan S, Slamecka J, Howze P, Thompson L, Finan M, Rocconi R, Owen L (2015). mRNA detection in living cells: a next generation cancer stem cell identification technique. Methods.

[CR21] Narcisi R, Cleary MA, Brama PA, Hoogduijn MJ, Tuysuz N, ten Berge D, van Osch GJ (2015). Long-term expansion, enhanced chondrogenic potential, and suppression of endochondral ossification of adult human MSCs via WNT signaling modulation. Stem Cell Rep.

[CR22] Narcisi R, Arikan OH, Lehmann J, Ten Berge D, van Osch GJ (2016). Differential effects of small molecule WNT Agonists on the multilineage differentiation capacity of human mesenchymal stem cells. Tissue Eng Part A.

[CR23] Pittenger MF, Mackay AM, Beck SC, Jaiswal RK, Douglas R, Mosca JD, Moorman MA, Simonetti DW, Craig S, Marshak DR (1999). Multilineage potential of adult human mesenchymal stem cells. Science.

[CR24] Prigodich AE, Seferos DS, Massich MD, Giljohann DA, Lane BC, Mirkin CA (2009). Nano-flares for mRNA regulation and detection. ACS Nano.

[CR25] Sacchetti B, Funari A, Michienzi S, Di Cesare S, Piersanti S, Saggio I, Tagliafico E, Ferrari S, Robey PG, Riminucci M, Bianco P (2007). Self-renewing osteoprogenitors in bone marrow sinusoids can organize a hematopoietic microenvironment. Cell.

[CR26] Seferos DS, Giljohann DA, Hill HD, Prigodich AE, Mirkin CA (2007). Nano-flares: probes for transfection and mRNA detection in living cells. J Am Chem Soc.

[CR27] Seftor EA, Seftor REB, Weldon D, Kirsammer GT, Margaryan NV, Gilgur A, Hendrix MJC (2014). Melanoma tumor cell heterogeneity: a molecular approach to study subpopulations expressing the embryonic morphogen nodal. Semin Oncol.

[CR28] Sivasubramaniyan K, Harichandan A, Schumann S, Sobiesiak M, Lengerke C, Maurer A, Kalbacher H, Buhring HJ (2013). Prospective isolation of mesenchymal stem cells from human bone marrow using novel antibodies directed against Sushi domain containing 2. Stem Cells Dev.

[CR29] Tian Y, Xu Y, Fu Q, Chang M, Wang Y, Shang X, Wan C, Marymont JV, Dong Y (2015). Notch inhibits chondrogenic differentiation of mesenchymal progenitor cells by targeting Twist1. Mol Cell Endocrinol.

[CR30] Yang J, Anholts J, Kolbe U, Stegehuis-Kamp JA, Claas FHJ, Eikmans M (2018). Calcium-binding proteins S100A8 and S100A9: investigation of their immune regulatory effect in myeloid cells. Int J Mol Sci.

