# Supplementary material for: Sorting living mesenchymal stem cells using a TWIST1 RNA-based probe depends on incubation time and uptake capacity
Source: Cytotechnology. 2019 Nov 14;72(1):37–45. doi: 10.1007/s10616-019-00355-w (PMC7002702; doi:10.1007/s10616-019-00355-w)
Supplement: Supplementary file 1 — Supplementary material 1 (PDF 458 kb) [file 10616_2019_355_MOESM1_ESM.pdf]

**SUPPORTIVE/SUPPLEMENTARY MATERIAL**

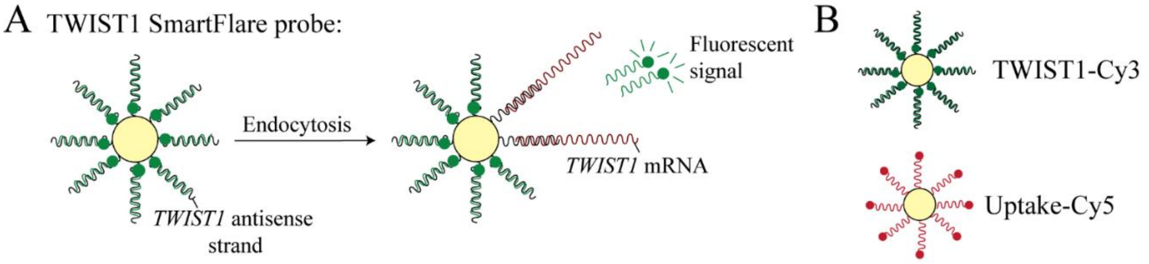


**Figure S1- Schematic overview of detection of TWIST1 expression by the SmartFlare probe.** Figure S1 is based on the figures in the SmartFlare manufacture user guide. (A) The TWIST1 SmartFlare probe exists of a gold particle with a *TWIST1* antisense strand attached to it. To this antisense strand a fluorescent reporter is bound, which is quenched by the gold particle. The probes enter the cells via endocytosis and if there is *TWIST1* mRNA in the cells, the fluorescent probe will be released and fluorescent. (B) The TWIST1-Cy3 probe is designed for specific detection of *TWIST1* mRNA. The Uptake-Cy5 is a control probe which is permanent fluorescent. The uptake-Cy5 fluorophore is not quenched because the fluorophore is located far from the gold particle.

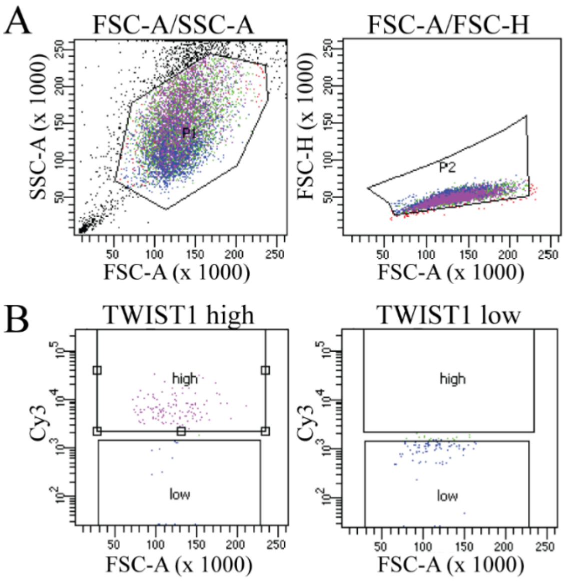


**Figure S2– FACS gating strategy to exclude cell debris and cell doublets.** (A) Cell debris are excluded by plotting FSC-A versus SSC-A (P1). Cell doubles are excluded by plotting FSCA versus FSC-H (P2). (B) Sorted populations were reanalyzed to test effective sorting.

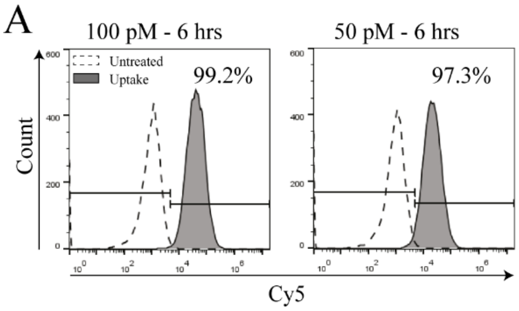


**Figure S3- SmartFlare probes are taken up by BMSCs after 6 hours**. (A) Uptake flow cytometry histograms of untreated BMSCs and BMSCs with 100 pM or 50 pM Uptake-Cy5 probe incubated for 6 hours. % shows percentage of Cy5 positive cells.

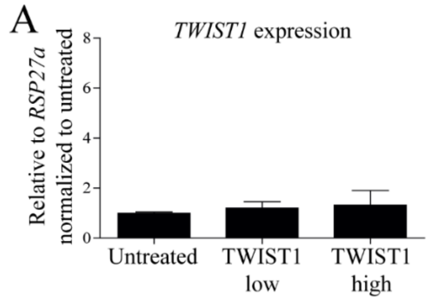


**Figure S4– RT-PCR results of *TWIST1$^{high}$* and *TWIST1$^{low}$* sorted BMSCs.** BMSCs are treated for 6 hours with 50 pM TWIST1-Cy3 probe and sorted based on TWIST1-Cy3 intensity (15% of the extremes) or left untreated. RT-PCR results, values represent the mean ±SD from triplicates.

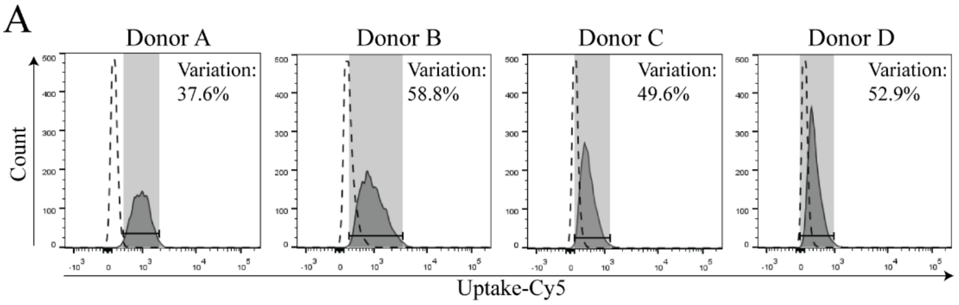


**Figure S5– Different MSC donors have a different probe uptake capacity**. Flow cytometry plots of BMSCs of four donors treated with Uptake-Cy5 probe and TWIST1-Cy3 probe for 6 hours show uptake variation (indicated in grey). Variation is coefficient of variation of the Uptake-Cy5 signal.

**Table S1- RT-PCR primers**

| **Gene** | **Primers** |
|---|---|
| *TWIST1* | Fw: 5’- GTCCGCAGTCTTACGAGGAG-3’<br>Rv: 5’- CCAGCTTGAGGGTCTGAATC-3’ |
| *RSP27a* | Fw: 5’-TGGCTGTCCTGAAATATTATAAGGT-3’<br>Rv: 5’-CCCCAGCACCACATTCATCA-3’ |
